# Blood-feeding patterns of *Anopheles* vectors of human malaria in Malawi: implications for malaria transmission and effectiveness of LLIN interventions

**DOI:** 10.1186/s12936-022-04089-7

**Published:** 2022-03-03

**Authors:** Rex B. Mbewe, John B. Keven, Themba Mzilahowa, Don Mathanga, Mark Wilson, Lauren Cohee, Miriam K. Laufer, Edward D. Walker

**Affiliations:** 1 Department of Physics and Biochemical Sciences, Polytechnic, University of Malawi, Blantyre, Malawi; 2 Department of Entomology, Michigan State University, East Lansing, MI 48824 USA; 3 Department of Public Health, University of California-Irvine, Irvine, CA USA; 4 Malaria Alert Center, College of Medicine, University of Malawi, Blantyre, Malawi; 5 Department of Epidemiology, School of Public Health, University of Michigan, Ann Arbor, MI USA; 6 Center for Vaccine Development and Global Health, University of Maryland School of Medicine, Baltimore, MD USA; 7 Department of Microbiology and Molecular Genetics, Michigan State University, East Lansing, MI USA

**Keywords:** *Anopheles*, Blood, Host, Malaria, Mosquitoes, Nets

## Abstract

**Background:**

Access to human hosts by *Anopheles* mosquitoes is a key determinant of vectorial capacity for malaria, but it can be limited by use of long-lasting insecticidal nets (LLINs). In Malawi, pyrethroid-treated LLINs with and without the synergist piperonyl butoxide (PBO) were distributed to control malaria. This study investigated the blood-feeding patterns of malaria vectors and whether LLINs containing pyrethroid and PBO led to a reduction of human blood feeding than those containing only pyrethroids.

**Methods:**

Mosquitoes were sampled inside houses from May 2019 through April 2020 by aspiration, pyrethrum spray catch, and light trap methods in two sites. One site (Namanolo, Balaka district) had LLINs containing only pyrethroids whereas the other (Ntaja, Machinga district) had LLINs with both pyrethroids and PBO. *Anopheles* species, their blood-meal host, and infection with *Plasmodium falciparum* were determined using PCR methods.

**Results:**

A total of 6585 female *Anopheles* were sampled in 203 houses. Of these, 633 (9.6%) were blood-fed mosquitoes comprising of 279 (44.1%) *Anopheles arabiensi*s, 103 (16.3%) *Anopheles gambiae* 212 (33.5), *Anopheles funestus*, 2 (0.3%), *Anopheles parensis* and 37 (5.8%) were unidentified *Anopheles* spp. Blood meal hosts were successfully identified for 85.5% (n = 541) of the blood-fed mosquitoes, of which 436 (81.0%) were human blood meals, 28 (5.2%) were goats, 11 (2.0%) were dogs, 60 (11.1%) were mixed goat-human blood meals, 5 (0.9%) were dog–human, and 1 was a mixed dog-goat. Human blood index (fraction of blood meals that were humans) was significantly higher in Namanolo (0.96) than Ntaja (0.89). Even though human blood index was high, goats were over-selected than humans after accounting for relative abundance of both hosts. The number of infectious *Anopheles* bites per person-year was 44 in Namanolo and 22 in Ntaja.

**Conclusion:**

Although LLINs with PBO PBO may have reduced human blood feeding, access to humans was extremely high despite high LLIN ownership and usage rates in both sites. This finding could explain persistently high rates of malaria infections in Malawi. However, this study had one village for each net type, thus the observed differences may have been a result of other factors present in each village.

**Supplementary Information:**

The online version contains supplementary material available at 10.1186/s12936-022-04089-7.

## Background

Malaria is endemic in Malawi with transmission occurring throughout the country, having generally greater transmission in the south than the north [1–3]. The nation-wide prevalence of infection for *Plasmodium falciparum* among 2 to 10 year old individuals, sampled from 2010 to 2017, declined from 29.4% in 2010 to 15.2% in 2017 but this change was uneven across the country’s 28 jurisdictional districts [4]. Despite these successes, which are largely attributed to reduction in transmission owing to the implementation of long-lasting insecticidal nets (LLINs) distributed throughout the country, Malawi remains a high malaria burden country with meso-endemic transmission [4]. In 2017, the country-wide incidence of malaria was 247 per 1000, with an estimated 7077 deaths [5].

One of the drivers of *Plasmodium* transmission is access to human blood meals by host-seeking female *Anopheles* mosquitoes. The level of access to human hosts is indicated by the relative proportion of human blood meals in a sample of blood-fed mosquitoes, often referred to as the human blood index (HBI). Therefore, selection of different vertebrate host species including humans for blood meals by *Anopheles* is a key determinant of vectorial capacity—a measure of malaria transmission—because it increases with HBI [6]. HBI is likely to be affected by use of different types of LLINs whose effectiveness is impacted by the extent of insecticide resistance in the *Anopheles* populations, and the relative availability of hosts [7, 8]. When vectors are susceptible to the pyrethroid insecticide used to treat the nets, HBI is reduced when LLINs are used. This happens because LLINs serve as barriers, preventing mosquitoes from accessing human hosts and also by killing those proportion of the mosquitoes that attempt to feed on humans sleeping under the nets. In contrast, those that feed on animals are not affected, resulting in higher animal blood-meal frequency. However, when pyrethroid resistance emerges in the vector populations, those mosquitoes that seek human hosts under the nets are not killed by the LLINs but live and acquire human blood meals from exposed humans (e.g., during times when humans are not under nets). Thus, insecticide resistance helps to increase the HBI, which means more humans are vulnerable to vector bites. Although the level of insecticide resistance was not investigated in this study, other studies have shown high level of pyrethroid resistance in the vector populations in the two study sites investigated here [9]. This has the potential to severely compromise the LLIN-based malaria control effort in Malawi because it would increase the likelihood of mosquito contact with human hosts. However, newer LLINs which combine pyrethroids with a synergist, piperonyl butoxide (PBO), have shown to be effective at restoring pyrethroid susceptibility [10, 11]. Despite presence of pyrethroid resistance in vector populations, the presence of LLINs with PBO, would decrease the HBI as the mosquito’s survival and access to human hosts is reduced.

In Malawi vector incrimination studies have identified members of the *Anopheles gambiae* complex and *Anopheles funestus* species assemblage as the most important malaria vectors and pyrethroid insecticide resistance have been reported [12–15]. However, only a single study has analysed the blood-feeding patterns and estimated the HBI of these vectors in the southern region of the country before LLINs were available [16]. Accordingly, whether the blood-feeding patterns has changed after the LLIN distribution campaign is unknown. This study had two objectives. The first objective was to expand on knowledge of blood-feeding patterns of malaria vectors in Malawi, by determining the host sources of their blood meals using molecular methods and conducting host selection analyses. Host selection is the tendency of vectors to feed on one host species relative to other available host species and is quantified by taking into account relative host abundance in the mosquitoes’ foraging area. The second objective was to determine if blood feeding on human hosts (i.e., HBI) is reduced where LLINs containing permethrin and the synergist PBO (i.e., Olyset Net Plus, Sumitomo Corporation, Tokyo, Japan) have been distributed compared to where standard, pyrethroid-only LLINs (i.e., Olyset Net, Sumitomo) have been distributed.

## Methods

### Study area

**Fig. 1:**
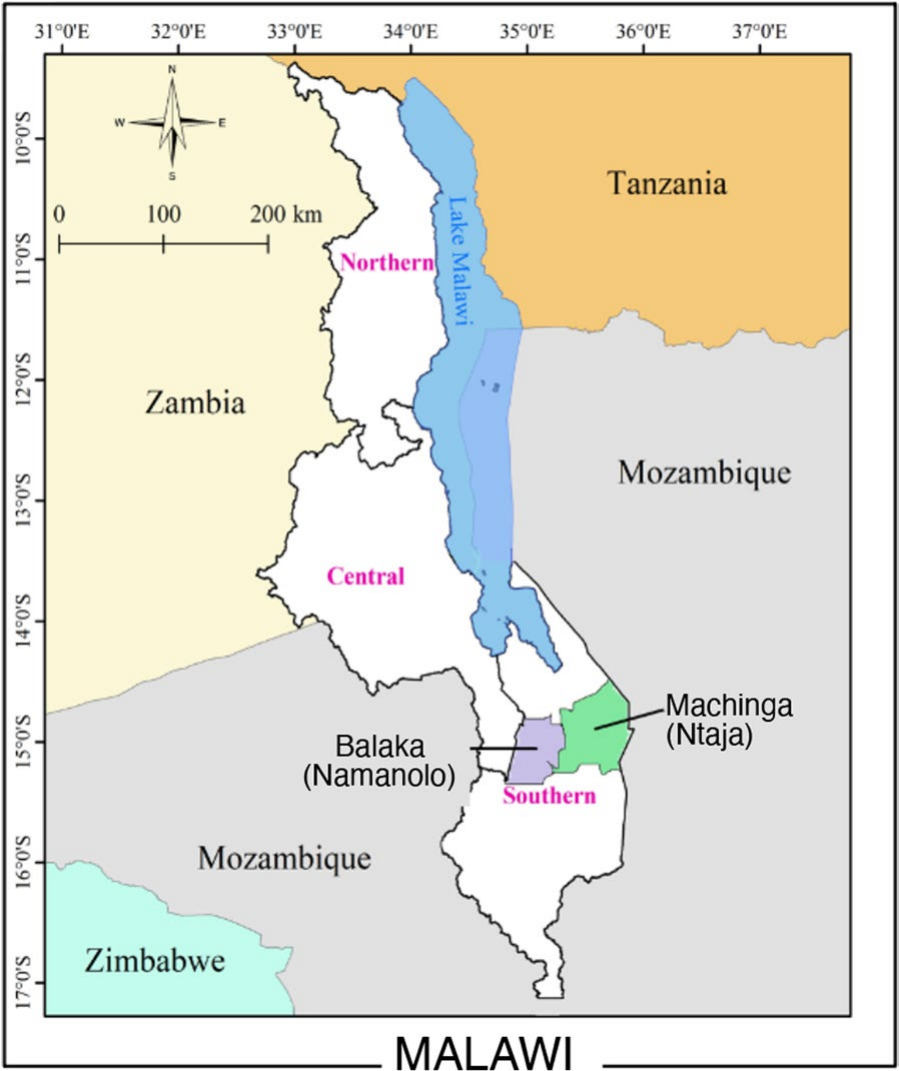
Map of Malawi showing the study sites in Balaka and Machinga districts

This study was conducted in two malaria-endemic districts of Malawi (Fig. 1), Balaka (14° 58′ 45″ S; 34° 57′ 20″ E) and Machinga (15° 10′ 6″ S; 35° 18′ 0″ E). These districts, like the whole of Malawi, have distinct wet and dry seasons where malaria proliferates especially in the rainy wet season [2]. In 2018, residents of Machinga received LLINs with PBO, whilst residents of Balaka received standard LLINs (National Malaria Control Programme, Government of Malawi, unpublished). Households in rural areas located within the catchment areas of Ntaja (Machinga) and Namanolo (Balaka) health centres were enrolled in the study. Household surveys determined that the rate of LLIN ownership was 92%, (n = 109 households) in Namanolo and 90% (n = 158 households) in Ntaja, and the nightly LLIN use rate was 75% (n = 109 households) in Namanolo and 74% (n = 158 households) in Ntaja (Malawi ICEMR project, unpublished).

### Mosquito sampling

Blood-fed *Anopheles* mosquitoes were sampled in houses, selected by the randomized cluster sampling method. All houses were sampled three times each period from May–June 2019, October–November 2019, and December 2019-January 2020. Beginning in February to April of 2020, only a subset of houses in each site were sampled once every 2 weeks. The mosquito samples for each visit were accumulated. The number of humans and goats in the houses was also counted, although dogs were too mobile to be counted reliably. Cattle were rare in the study area and scored as absent. Mosquitoes were sampled using mouth and battery-powered aspirators, standard miniature Center for Disease Control light traps (Model 512; John W. Hock Company, Gainesville, Florida, USA) and pyrethrum spray catches [15, 16]. Mosquitoes were morphologically identified [17] into the *An. gambiae* complex, the *An. funestus* group, or other *Anopheles* species. Mosquitoes were stored individually in tubes with silica gel desiccant and kept under cool temperature in the laboratory.

### Molecular identification of mosquito species

For each mosquito, the abdomen was separated from the anterior body part (head and thorax) using sterile, cross-contamination-proof technique. Genomic DNA was extracted from the abdomen and head-thorax separately using DNeasy Blood & Tissue Kit (Product number: 69582; Qiagen, Valencia, CA, USA) following the manufacturer’s protocol. Mosquitoes of the *An. gambiae* complex were identified to one of two species, *An. gambiae* or *Anopheles arabiensis*, using a published multiplex quantitative polymerase chain reaction (PCR) method [18]. Mosquitoes of the *An. funestus* species assemblage were identified to one of three species, *An. funestus*, *Anopheles parensis* and *Anopheles vaneedeni* using another multiplex quantitative PCR as follows. A universal primer pair that amplifies a region of the internal transcribed spacer region 2 of the ribosomal ribonucleic acid gene of members of the *An. funestus* species assemblage was designed along with an oligonucleotide probe specific to *An. parensis*. The probes specific to *An. funestus* and *An. vaneedeni* was obtained from a published source [19] and bind to the DNA locus amplified by the universal primer pair. Nucleotide sequence of the universal primers and the probes specific to each of the three vector species are shown in Table 1. The probes were labelled with the reporter dye FAM, VIC or ABY and quencher dye QSY (Table 1). After performing optimization tests involving tenfold dilution series of positive DNA control of the three species, the optimum PCR mixture (10 µl reaction volume) consisted of 1 × TaqMan Universal Master Mix (Product number: 4304437; Thermo Fisher Scientific, Waltham, MA, USA), 0.6 µM of each primer, 0.4 µM of each probe, and 2 µl of mosquito DNA. The reactions were performed on QuantStudio 7 Flex PCR system (Applied Biosystems, Foster City, CA, USA) using the following cycling conditions: one cycle of 50 °C for 2 min and 95 °C for 10 min, 40 cycles of 95 °C for 15 s and 60 °C for 1 min. PCR sensitivity was one target gene copy/µl sample and efficiency was > 90%. Positive and negative DNA controls were included in each experiment. Samples with amplification threshold cycles ≥ 38 were considered inconclusive and, therefore. negative. The PCR results were visualized with QuantStudio software (version 1.3).

**Table 1:** Primers and probes for three species within the *An. funestus* species assemblage and their blood-meal hosts

Organism	Nucleotide sequence (5′-3′)
*An. funestus*	Forward: AGA ACA CTA TGG CGA GCA GC
	Reverse: TTA CGA CGG ATA CGG TCA ACG
	Funestus probe: FAM-CAT GGG GAA ATT CAA TCG AAA ACC TCT-QSY
	Parensis probe: ABY-TGG CGT GCT CGG AAC CTA GC-QSY
	Vaneedeni probe: VIC-CGT TGT GAA AAA TGG AGA TTC ATT TGA AAA CC-QSY
Human	Forward: GGC CTG TTC CTC CCT TAT TT
	Reverse: TAC ACA GGG CTT CCG AGT
	Probe: FAM-ATG GAG TCT GTG TTC CCT GTG ACC-QSY
Goat	Forward: TAG GCG CCA TGC TAC TAA TTC
	Reverse: GAG TGG ATT TGC TGG GAT ATA G
	Probe: VIC-ATT CAC ACC CGA CCT ACT CGG AGA-QSY
Dog	Forward: TGG ACA AAG CAA CCC TAA CA
	Reverse: CCG GTT TCG TGT AGA AAT AGG A
	Probe: ABY-TCA TCC TCC CTT TCA TCA TCG CAG C-QSY
Vertebrates	Forward: CCC CTC AGA ATG ATA TTT GTC CTC A
	Reverse: CCA TCC AAC ATC TCA GCA TGA TGA AA

### Molecular identification of blood-meal hosts

Individual mosquito abdominal DNA was first tested for human blood meal using a uniplex quantitative PCR method with primers and probe (Table 1) that target a region of intron 1 of the nuclear tyrosine hydroxylase gene as described in Keven et al. [20]. Samples that did not react with the human probe were subjected to a standard PCR to amplify the vertebrate mitochondrial cytochrome B gene using a generic primer pair (Table 1) developed by Boakye et al. [21] and used in several blood-meal studies [22–24]. The standard PCR reaction mixture (25 µl volume) consisted of 10 mM Tris at pH 8.3, 50 mM KCl, 1.5 mM MgCl_2_, 0.01% gelatin, 1.0 mM dNTP, 0.5 units of Taq polymerase, 50 pmol of each primer, and approximately 10 ng of DNA template. The reaction condition included one cycle of 95 °C for 5 min (initial denaturation) followed by 35 cycles of 95 °C for 1 min (denaturization), 57 °C for 1 min (annealing) and 68 °C for 1 min (extension), followed by one cycle of 68 °C for 5 min (final extension). The PCR products were visualized with 2% agarose gel electrophoresis and amplicons of positive samples were purified using QIAquick PCR purification kit (Product number: 2810; Qiagen) following the manufacturer’s protocol. The nucleotide sequence of the amplicons was determined by direct sequencing and the sequences were subjected to BLAST (Basic Local Alignment Search Tool) analysis to search for potential matches to the available vertebrate host cytochrome B gene sequences in GenBank database. A sequence similarity of 97% or higher was used as the cut-off for an acceptable match, based on literature [22, 25, 26].

The different non-human host species identified in the mosquito blood meals based on the results of BLAST searches aided the development of new primers and probes specific to those hosts; dogs and goats were the only hosts apart from humans identified by BLAST searches (see Results). As with humans, the primers and probe for dogs were adopted from a published source, but those for goats were designed and validated in the current study using the same procedure for humans and dogs [20]. A multiplex quantitative PCR containing the probes for all three host species was optimized and performed on all the blood-meal samples. The purpose of this second quantitative PCR was to detect presence of mixed blood meals as well as to confirm the results of the previous uniplex human quantitative PCR. The optimized multiplex, quantitative PCR mixture (10 µl reaction volume) consisted of 1 × TaqMan Universal Master Mix (Product number: 4304437; Thermo Fisher Scientific), 0.5 µM of each primer, 0.25 µM of each probe, and 2 µl of DNA. PCR cycling condition (QuantStudio 7 Flex PCR System) was the same as described for *An. funestus* group above. Positive and negative DNA controls were included in each experiment. Samples with amplification threshold cycles ≥ 38 were considered inconclusive and therefore negative.

### Molecular detection of *P. falciparum* sporozoites

DNA from the head-thorax of each mosquito was tested for presence of *P. falciparum* using a quantitative PCR method described in Keven et al. [27]. *Plasmodium* sporozoites inhabit the salivary glands in the head and thorax of mosquitoes [28, 29]. Therefore, by analysing the abdomen detached body part (i.e., head-thorax) of the mosquitoes, the PCR-positive samples were more likely to carry sporozoites than other stages, including human stages, of the parasites which are found in the mosquito abdomen [28, 29].

### Data analysis

Mosquitoes whose blood meal hosts were not identified were excluded from any analysis involving blood meal data. Mosquitoes that had fed on one species of host were classified as single-host blood meals. If the blood-meal analysis revealed two or more species of vertebrate hosts, then the blood meals were classified as mixed blood meals. An *Anopheles* population is mosquitoes of a particular *Anopheles* species from a particular area. When calculating the HBI of an *Anopheles* population, human-fed mosquitoes included both single human blood meals as well as human-animal mixed blood meals. Differences in HBI between study sites with different types of LLINs were analysed by Chi-square analysis of a contingency table. To test for variation in the propensity of the three main malaria vector species to feed on human, animal and human-animal mix blood-meal types, a 3 × 3 contingency table with chi-square analysis was carried out, and the percentage deviations of observed from expected frequencies were calculated. Host selection of a vector population was quantified using theta statistic (θ = π_1_/π_2_), which tested whether the ratio (θ) of the proportion of mosquito blood meals that fed on the host of interest (π_1_) and proportion of all the hosts of interest in the mosquito foraging area (π_2_) is different from unity [30]. A host species was considered over-selected by the vector population if theta was significantly greater than 1.0 or under-selected if theta was significantly less than 1.0. A host species was considered to be fed on by the mosquitoes in proportion to its relative abundance in the village if theta was not significantly different from 1.0. The theta and Chi-square analyses were performed using the *ci.prat.ak* and *chisq.test* functions of the package *asbio* and *stats* package, respectively, in R software version 3.4.2 (https://www.r-project.org/).

The sporozoite rate (SR) was estimated as the proportion of mosquito heads-thoraces that tested positive for *Plasmodium*. The entomological inoculation rate (EIR) can be estimated indirectly from samples obtained by indoor resting mosquitoes, and also directly from samples obtained by the human landing catch method [31]. In the current study, samples from indoor resting collections were used to indirectly calculate the EIR using the formula EIR = (M*SR*HBI)/N, where M is the mean number of blood-fed mosquitoes per house and N is the mean number of human occupants per house per night. SR and HBI are defined above. The EIR estimated using the above formula is the number of infectious bites per person-night. Annual EIR (number of infectious bites per person-year) was estimated by multiplying the nightly EIR by 365, which is the number of days in a year.

## Results

### Species composition

Mosquitoes were collected in 203 houses, yielding 6,585 female *Anopheles* mosquitoes including 633 (9.6%) blood-fed ones. Blood-fed mosquitoes were collected in only 50 of the 203 houses, and consisted of the following species as determined by the combination of morphological and molecular analyses: *An. arabiensis* (Overall: 279, Namanolo: 242, Ntaja: 37), *An. funestus* (Overall: 212, Namanolo: 110, Ntaja: 103)*, An. gambiae* (Overall: 103, Namanolo: 32, Ntaja: 70), and *An. parensis* (Overall: 2, Namanolo: 1, Ntaja: 1). Thirty-seven were not identified to species by morphological or molecular means.

### Blood-meal hosts

**Fig. 2:**
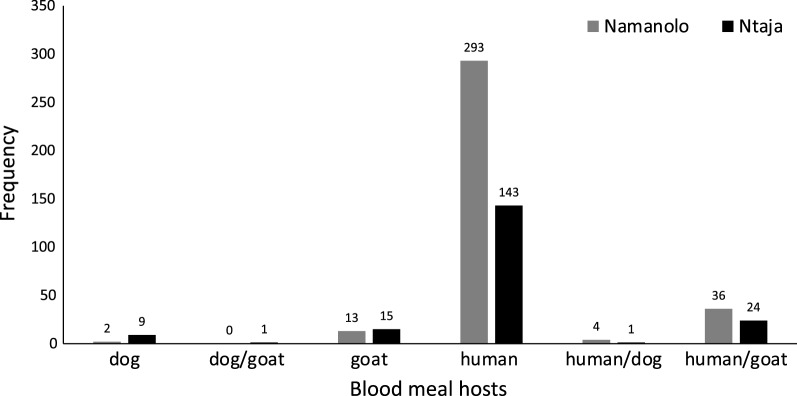
Blood meal identification to vertebrate host, including mixed meals, for *Anopheles* mosquitoes in the two study sites

Of the total blood-fed *Anopheles* mosquitoes (n = 633, all sampling methods combined), the blood-meal host of 541 (85.5%) were successfully identified either by qPCR or direct sequencing (Fig. 2 and Table 2). The remaining 92 (14.5%) were either non-reactors in PCR reactions (n = 42), or amplicons generated by standard PCR failed to match any feasible host (n = 50) due to poor sequence quality. Of the 541 mosquitoes whose blood-meal host was successfully identified, 436 (81.0%) were solely human blood meals, 28 (5.2%) were solely goat blood meals, 11 (2.0%) were solely dog blood meals, and mixed blood meals were: 1 (0.2%) dog–goat, 5 (0.9%) dog–human, and 60 (11%) goat–human (Fig. 2 and Table 2). The number of blood-fed *Anopheles* mosquitoes collected by the different sampling methods is presented in Additional file 1: Table S1.

**Table 2:** Blood-meal sources of *Anopheles* mosquito species in the two study sites. Values outside parenthesis are number of mosquitoes and inside parenthesis are the proportion of row totals

Site	Vector	Human	Dog	Goat	Human/dog	Human/goat	Dog/goat
Namanolo	*An. arabiensis*	185 (84.9)	2 (0.9)	7 (3.2)	3 (1.4)	21 (9.6)	0 (0)
	*An. funestus*	75 (87.2)	0 (0)	2 (2.3)	0 (0)	9 (10.5)	0 (0)
	*An. gambiae*	24 (80)	0 (0)	1 (3.3)	0 (0)	5 (16.7)	0 (0)
	*An. parensis*	0 (0)	0 (0)	1 (100)	0 (0)	0 (0)	0 (0)
	*Anopheles spp.*	9 (69.2)	0 (0)	2(15.4)	1 (7.7)	1 (7.7)	0 (0)
Ntaja	*An. arabiensis*	16 (43.2)	6 (16.2)	4 (10.8)	1 (2.7)	9 (24.4)	1 (2.7)
	*An. funestus*	75 (88.2)	3 (3.5)	1 (1.2)	0 (0)	6 (7.1)	0 (0)
	*An. gambiae*	47 (79.7)	0 (0)	5 (8.5)	0 (0)	7 (11.8)	0 (0)
	*An. parensis*	0 (0)	0 (0)	0 (0)	0 (0)	1 (100)	0 (0)
	*Anopheles spp.*	5 (45.5)	0 (0)	5 (45.5)	0 (0)	1 (9.0)	0 (0)

Humans were the most frequently identified blood-meal host for all three of the most abundant *Anopheles* species (*An. arabiensis*, *An. funestus* and *An. gambiae*). Mixed blood meals comprising human and goat were present for all three mosquito species and ranged from 7.1 to 11.7% among them. Blood meals identified solely from goats ranged from 1.4 to 5.8% and were also found in all three species. Dog-only, and mixed dog–human or dog–goat blood meals, were present in the sample, but uncommon. Differences in human or animal (goat and dog) and mixed (human-animal) host feeding by these three species, and the percentage deviations of observed from expected frequencies are shown in Table 3. Although the Chi-square test was not significant (χ^2^ = 6.4, df = 4, p = 0.17), the percentage deviation values suggested that *An. arabiensis* tended to feed on the animal hosts more so than did *An. gambiae* and *An. funestus,* and *An. funestus* tended to underutilize the animal hosts compared to *An. gambiae* and *An. arabiensis*. Additionally, *An. funestus* tended to have fewer human-animal mixed blood meals compared to the other species (Table 3).

**Table 3:** Percent deviation of observed blood-meal frequencies (data from both sites combined) from those expected by Chi-square analysis for the three primary vector species

Species	Human	Animal	Human-animal mix
*An. arabiensis*	− 4.2	+27	+15.5
*An. gambiae*	− 1.8	+7	+8.7
*An. funestus*	+7.0	− 42.5	− 26.9

### Host selection

**Fig. 3:**
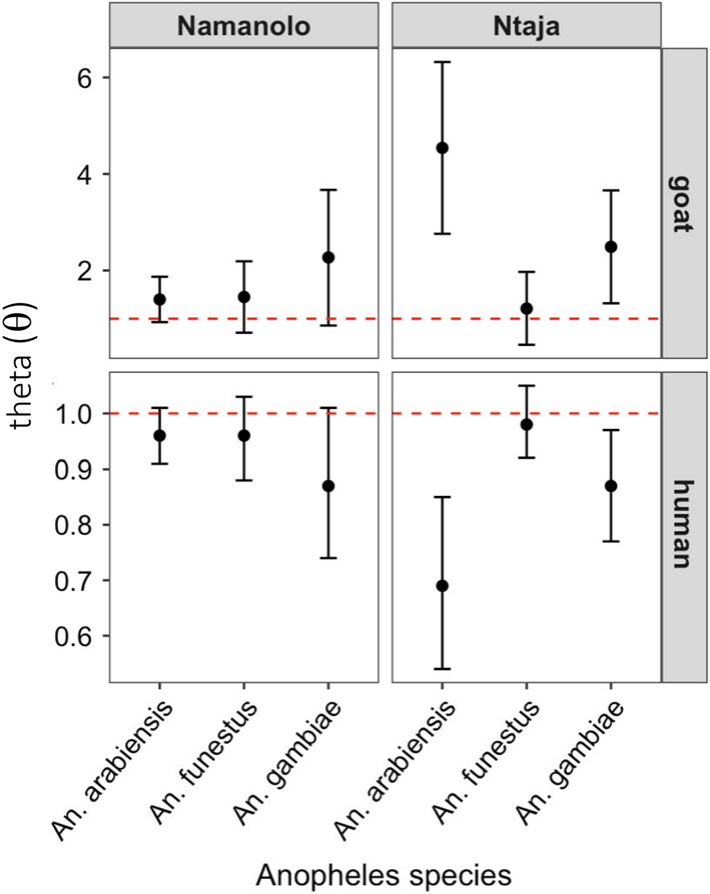
Results of theta statistical analysis of host selection tendency for goats relative to humans (top panels) and humans relative to goats (bottom panels) of *Anopheles* species in the two study sites. Black shaded circles are theta values with 95% CI bars. Red dotted line at 1.0 represents random selection in relation to availability of both hosts. The 95% CI bars represent deviation from random selection pointing to over-selection (theta significantly > 1.0) or under-selection (theta significantly < 1.0)

Out of 50 households where blood-fed mosquitoes were collected, there was a total of 233 individual humans (142 in Ntaja and 91 in Namanolo); 30 (60%) of these households reported keeping goats and kept goats indoors overnight. Fifteen houses were in each study village. Householders reported a total of 104 goats, 49 in Namanolo (average per house, 3.3) and 55 in Ntaja (average per house, 3.7). The other 20 households did not report keeping goats. The host abundance data reported above was used together with the blood-meal data to estimate host selection of vectors by theta statistical analysis. The results (Fig. 3) show that in Ntaja, despite overwhelmingly large number of humans compared to goats, *An. arabiensis* and *An. gambiae* tended to over-select goats and under-select humans, whilst *An. funestus* selected these two host species in proportion to their relative abundance in the village. In Namanolo, by contrast, all three vector species selected both hosts in proportion to their relative abundance, although there was a nonsignificant tendency for over-selection of goats compared to humans despite overwhelmingly large number of humans compared to goats.

### Effect of LLIN types on HBI

The three abundant mosquito species (data from both villages combined) had similar HBI: 0.97 (n = 171) for *An. funestus*, 0.94 (n = 89) for *An. gambiae* and 0.92 (n = 255) for *An. arabiensis*. The HBI among these species was not significantly different (χ^2^ = 0.11, df = 2, P = 0.946). To test the effect of LLIN types on HBI, blood-meal data from all three species within each study site were combined. The HBI in Namanolo was 0.96 (n = 348) and in Ntaja was 0.87 (n = 193). The result of Chi-square analysis showed that HBI was significantly higher in Namanolo, where pyrethroid only LLINs were used, compared with Ntaja where pyrethroid plus PBO LLINs were used (χ^2^ = 12.3, df = 1, P = 0.0004).

### Sporozoite and entomological inoculation rates

PCR tests of *P. falciparum* sporozoite infection in the head-thorax of *Anopheles* mosquitoes (regardless of species) from all villages combined gave an overall SR of 0.16 (n = 633). The SR for each of the three primary species in each study site are shown in Table 4. The SR for all three primary species combined in Namanolo was 0.15 (n = 385) and in Ntaja was 0.19 (n = 209). The blood-fed mosquitoes were collected in 203 houses with 1,106 occupants (Namanolo: 97 houses, 488 occupants; Ntaja: 106 houses, 618 occupants). These data were used to calculate N and M and together with HBI and SR were used to estimate EIR (see formula in Methods). The overall EIR (both sites combined) was equal to (633/203*0.16*0.94)/(1106/203) or 0.09 infectious bites per person-night, equivalent to 32.9 infectious bites per person-year. For Namanolo, the EIR was equal to (410/97*0.15*0.96)/(488/97), or 0.12 infectious bites per person-night (44 infectious bites per person-year). For Ntaja, the EIR was equal to (223/106*0.19*0.89)/(618/106) or 0.06 infectious bites per person-night (22 infectious bites per person-year).

**Table 4:** Species specific EIR and SR in the two study sites

	Namanolo		Ntaja	
Vector	SR	EIR	SR	EIR
*An. arabiensis*	0.18 (n = 244)	0.15	0.15 (n = 39)	0.38
*An. funestus*	0.09 (n = 109)	0.7	0.16 (n = 103)	0.05
*An. gambiae*	0.16 (n = 32)	0.13	0.25 (n = 67)	0.08

EIR measured in infectious bite per person-night. For SR, n is the number of mosquitoes that were tested for sporozoite infection

## Discussion

Indoor mosquito sampling of rural houses in the two study districts of southeastern Malawi revealed three major malaria vector species. This finding was consistent with past studies in Malawi and southern Africa [12, 13, 15]. The results here provide key malariologic transmission indices (HBI, EIR) that demonstrate the vulnerability of humans to bites of vector *Anopheles* mosquitoes, despite the presence and use of LLINs as the primary anti-malaria intervention. Although no species was numerically dominant, *An. funestus* and *An. gambiae* were relatively more common in Ntaja and *An. arabiensis* was more common in Namanolo. By contrast, a study that conducted indoor collections of mosquitoes at other locations close to both Namanolo and Ntaja found *An. funestus* to be dominant, while *An. arabiensis* was next in abundance and *An. gambiae* was uncommon [9]. The populations in that study exhibited resistance to the synthetic pyrethroid deltamethrin, with 38% mortality in World Health Organization bioassays for *An. funestus* group and 53% mortality for *An. gambiae* complex (probably *An. arabiensis*) [9]. Despite these variations in mosquito species abundances between sites, *An. funestus* and *An. gambiae* are generally considered epidemiologically more important than *An. arabiensis* due to their well-documented anthropophilic and endophilic behaviours [32–35]. This study found that when mosquitoes entered houses, there was a high rate of human blood feeding by all three vector species, regardless of these variable phenotypes.

The finding that human blood comprised most blood meals in unmixed conditions, and with goat blood meal being more frequent than dog, is not surprising because it was commonly observed that people kept goats indoors in special rooms at night, probably for protection against theft, while dogs were left outside as guard dogs. This may explain the higher number of goat blood meals compared to dog blood meals. Killeen et al. [32] demonstrated by modeling that there is a relationship between host availability and the amount of time that African *Anopheles* malaria vectors spend seeking blood meals; by inference, hosts that require less time to locate will be fed upon more frequently. Orsborne et al. [33] reached a similar conclusion, emphasizing that local host availability even for known anthropophilic malaria vectors, is a powerful driver for host selection. In Malawi, there have been no previous studies that consistently quantified relative availability of potential blood-meal hosts. The high prevalence of human host blood feeding by *Anopheles* species observed here is consistent with a study from southern Malawi conducted in 2002, in which blood meals were nearly entirely from humans and secondarily from bovines [16]. In northern and southern Zambia, similar high human host selection (> 90%) and comparatively lower goat selection (< 5%) by *An. gambiae.* and *An. funestus* were observed [36, 37]. In contrast, the dominant blood-meal source of malaria vectors around Lake Victoria in western Kenya was humans for *An. gambiae* and *An. funestus* but for *An. arabiensis* was predominantly bovine [38] or equally bovine and human [39]. This study approached the problem of variation in host selection of these often behaviourally stereotyped species through application of theta statistical analysis which takes into account the relative abundance of host species in the mosquito foraging area [30].

The frequency of human blood meals detected in the study sites was high and could be attributed to several factors, in particular bed net use practices, such as incorrect use as well as inconsistent nightly use. In both sites, the nightly use rates were slightly lower than net ownership rates. The lower HBI in Ntaja (0.89) compared to Namanolo (0.96) could be due to widespread use of PBO-containing LLINs in Ntaja, which have been shown to be more effective than conventional LLINs against pyrethroid-resistant *Anopheles* populations [14]. Lindsay et al. [40] have suggested that the underlying mechanism of PBO-containing LLINs may simply be that they are more toxic, rather than merely overcoming insecticide resistance. Regardless, other randomized field trials in Tanzania and Uganda have shown significantly lower human infection prevalence where LLINs with PBO were distributed [10, 11]. The entomological mediators of these reductions were likely due to lower transmission intensity. Here, the estimated annual EIR was lower in Ntaja (22 infectious bites per person-year) than Namanolo (44 infectious bites per person-year), however, this difference could be due to site-specific characteristics as opposed to different LLIN types.

In the only other study analysing blood meals of *Anopheles* vectors in Malawi, conducted in Chikwawa district (southern Malawi) during 2002 prior to any mass distribution of insecticide-treated nets, most blood meals were from humans, with relatively few coming from bovine or mixed human-bovine feeding [16]. The 2002 HBI estimates for the three dominant malaria vector species at that time were similar to what was found in the present 2019–2020 study, despite there now being a long history of malaria control and LLIN use in the intervening period. The species-specific HBI estimates for 2002 vs. 2019–2020 were: *An. arabiensis*, 0.85 vs. 0.92; *An. gambiae* 0.99 vs. 0.94; and *An. funestus* 0.99 vs. 0.97. However, the estimated annual EIR in the present study (33 infectious bites per person-year) was lower than that reported previously (183 infectious bites per person-year) [16]. Both studies used PCR-based detection of sporozoite infection in the head-thorax of individual mosquitoes, although SR was lower in the 2002 investigation (0.049) compared to the present study (0.16). However, indoor mosquito density was lower at the present study sites, thereby reducing the EIR. Another more recent study in Chikwawa, done during the implementation of a community-based control program, showed that 4 of 91 *Anopheles* (SR, 0.044) tested by PCR were positive for *P. falciparum* infection during the rainy season, with an estimated EIR of 13.5 infectious bites per person-year [41], suggesting a reduced EIR in that region. Considering the long dry season in Malawi, these are likely overestimates when annualized, and could be half these values.

Molecular-based approaches to blood-meal analysis to detect vertebrate host feeding have advanced (e.g., [20, 38, 42]) since the review of this topic by Kent [25]. At the forefront of this advance has been development of quantitative PCR methods using host-specific probes by either TaqMan or SYBR green detection [20, 43]. However, host species-specific probes in multiplex quantitative PCR targeting *Anopheles* bloodmeal hosts were developed only recently [20]. The use of species-specific probes, designed within a quantitative PCR format here, favored the detection of single and multiple (i.e., mixed) blood meals in this study. By screening all blood meals for human blood by quantitative PCR and then analysing by standard PCR, amplicon sequencing and BLAST search matching those blood meals not reacting to the human probe, it was possible to reveal the narrow breadth of dominant hosts being utilized by the *Anopheles* community. The development of quantitative PCR probes for the animal hosts helps determine the frequency of mixed blood feeding.

The host selection analysis showed that, in one site (Ntaja), two of the *Anopheles* species fed more often on goats than humans in proportion to availability of these hosts. *An. arabiensis* and *An. gambiae* over-selected goats and under-selected humans, while *An. funestus* selected the two hosts about equally (i.e., randomly) in proportion to their availability. These results are not surprising. Whilst *An. arabiensis* is reportedly more zoophilic, *An. funestus* is more anthropophilic [44]. Plasticity and or opportunistic tendencies for blood host selection have been observed in various *Anopheles* species [7, 32–35]. The explanation to the over-selection of goats in indoor mosquito samples is consistent with goats being kept indoors at night, providing ready access to mosquitoes seeking hosts indoors, and to LLINs deployed indoors which could limit access to sleeping humans and divert mosquitoes to indoor goats [32]. This study will prompt more research in blood-meal studies in Malawi to document the range of blood-meal hosts, especially those involving goat blood meal which is uncommonly reported in literature despite widespread presence of goats in villages of sub-Saharan Africa.

The findings of this investigation suggest important implications for *Plasmodium* transmission and malaria control. Multiple host feeding by some *Anopheles* females might allow for increased survival and reproduction [42, 45]. The presence of multiple malaria vector species that successfully obtain human blood meals could lead to an increased *Plasmodium* transmission by increasing the basic reproductive number [36, 37]. More widespread use of LLINs, particularly with PBO, could help reduce transmission, but this intervention alone is unlikely to reduce malaria incidence in this meso-endemic setting to acceptable levels where elimination can be contemplated. Residual *Plasmodium* transmission and weakened intervention efforts [46] are likely to persist into the future.

A limitation of this study was that it involved only indoor collections and hence would not capture mosquito host-seeking nor resting behaviour outdoors. Future studies should incorporate this element as well as the significance of insecticide susceptibility and resistance in relationship to host selection.

## Conclusion

This study has shown that, in southern Malawi, human blood comprises the bulk of the blood meals of the three primary *Anopheles* vector species, yet dog and goat blood meals are also present and commonly mixed with that of humans. Host selection analysis revealed that goats were over-selected compared to humans at the site where PBO LLINs were in use, even though human blood meals were by far the most common at both sites. This observation could be due to PBO LLIN usage. The presence of mixed blood meals may reveal the adaptability of these vectors to switch hosts to obtain a full blood meal, possibly reducing effectiveness of malaria control interventions. Generally, the frequent use of humans as a blood-meal source, observed here, elevates the HBI and consequently the EIR, likely sustaining malaria transmission. This study shows that pyrethroid-based LLINs containing PBO may have reduced mosquito-human contact as the HBI was significantly lower at the site where people used the PBO nets. However, a multi-site study design is needed to confirm this observation.

## Supplementary Information

**Additional file 1: Table S1.** Number of blood-fed mosquitoes by blood-meal hosts, sampling methods and study sites. Values in parenthesis are percentages of column totals.

## Data Availability

Data supporting the conclusions of this article are included within the article and its additional files.

## Abbreviations

BLAST: Basic local alignment search tool
DNA: Deoxyribonucleic acid
EIR: Entomological inoculation rate
HBI: Human blood index
ICEMR: International Center for Excellence in Malaria Research
LLINs: Long-lasting insecticidal nets
PBO: Piperonyl butoxide
PCR: Polymerase chain reaction
SR: Sporozoite rate
USA: United States of America
