# Supplementary material for: Blood-feeding patterns of Anopheles vectors of human malaria in Malawi: implications for malaria transmission and effectiveness of LLIN interventions
Source: Malar J. 2022 Mar 3;21:67. doi: 10.1186/s12936-022-04089-7 (PMC8892392; doi:10.1186/s12936-022-04089-7)
Supplement: Supplementary file 1 — Additional file 1: Table S1. Number of blood-fed mosquitoes by blood-meal hosts, sampling methods and study sites. Values in parenthesis are percentages of column totals. [file 12936_2022_4089_MOESM1_ESM.docx]

Table S1. Number of blood-fed mosquitoes by blood-meal hosts, sampling methods and study sites. Values in parenthesis are percentages of column totals.

|  | Namanolo | | | Ntaja | | |
| --- | --- | --- | --- | --- | --- | --- |
| Blood meal | Aspiration | CDCLT | PSCs | Aspiration | CDCLT | PSCs |
| Human | 0 (0) | 126 (66.3) | 164 (74.2) | 0 (0) | 36 (53.7) | 106 (68.4) |
| Dog | 0 (0) | 2 (1.1) | 0 (0) | 0 (0) | 1 (1.5) | 8 (5.2) |
| Goat | 0 (0) | 6 (3.2) | 8 (3.6) | 0 (0) | 12 (17.9) | 3 (1.9) |
| Mixed | 0 (0) | 14 (7.4) | 26 (11.8) | 0 (0) | 10 (14.9) | 16 (10.3) |
| Unidentified | 0 (0) | 42 (22.1) | 23 (10.4) | 0 (0) | 8 (11.9) | 22 (14.2) |

Note: CDCLT; Center for Disease Control miniature light trap, PSCs; pyrethrum spray catches
